# Severe acute respiratory infection risk following glucocorticosteroid treatment in uncomplicated influenza-like illness resulting from pH1N1 influenza infection: a case control study

**DOI:** 10.1186/s12879-019-4669-9

**Published:** 2019-12-26

**Authors:** Xuesen Xing, Shixiong Hu, Meihua Chen, Faxian Zhan, Huihui Liu, Zhang Chen, Hengjiao Zhang, Ge Zeng, Qiaohua Xu, Hong Zhang, Man Liu, Honghui Liu, Lidong Gao, Lijie Zhang

**Affiliations:** 1 Hubei Provincial Center for Disease Control and Prevention, Wuhan, Hubei Province China; 2 Chinese Field Epidemiology Training Program, Chinese Center for Disease Control and Prevention, 27 Nanwei Road, Xicheng District, Beijing, 100050 China; 3 Hunan Provincial Center for Disease Control and Prevention, Changsha, Hunan Province China; 4 Wuhan No. 1 Hospital, Wuhan, Hubei Province China

**Keywords:** pH1N1 influenza, Severe acute respiratory infection, Glucocorticosteroid treatment

## Abstract

**Background:**

Current studies regarding glucocorticosteroid treatment of influenza have only estimated risk of critical illness or death which can be easily confounded by timing of treatment administration. We used severe acute respiratory infection (sARI) as an endpoint and investigated risk associated with receiving glucocorticosteroids before sARI onset.

**Methods:**

sARI cases were defined as influenza-like illness (ILI) with pH1N1 infection and respiratory distress. Controls were defined as pH1N1 cases other than sARI and randomly selected from the community. We compared glucocorticosteroids and other medications used before sARI onset using a matched case control study adjusted for age group as well as underlying disease. Time-dependent risk and dose responses at different time periods over the course of sARI cases were also examined.

**Results:**

Of the sARI cases, 34% received glucocorticosteroids before sARI onset compared to 3.8% of controls during equivalent days (OR_M-H_ = 17,95%CI = 2.1–135). Receiving glucocorticosteroids before sARI onset increased risk of developing subsequent critical illness or death (OR_M-H_ = 5.7,95%CI = 1.6–20.2), and the OR_M-H_ increased from 5.7 to 8.5 for continued glucocorticosteroid use after sARI onset. However, only receiving glucocorticosteroids after sARI onset did not increase risk of severe illness (OR_M-H_ = 1.1,95%CI = 0.3–4.6). Each increase in glucocorticosteroids dose of 1 mg/kg/day before sARI onset resulted in an increase of 0.62 (*R*^2^ = 0.87) in the pMEWS score at the time of sARI onset.

**Conclusions:**

Early glucocorticosteroid treatment increased risk of sARI and subsequent critical illness or death; however, only receiving glucocorticosteroids after sARI onset did not increase risk of severe illness.

## Background

The rapid global spread of influenza pandemics has been well documented and has resulted in one of the greatest health-related catastrophes documented in humans. The influenza pandemic of 1918–1919 was estimated to have resulted in over 500 million cases and 50 million deaths worldwide [1]. Although the case fatality ratio associated with the 2009 pandemic influenza A (pH1N1) was lower than that previously reported (0.03% versus 2.5% in the 1918–1919 pandemic) [2], worldwide clinical data from the pH1N1 pandemic revealed that more than one-fifth of hospitalized individuals experienced severe disease that required intensive care unit admission [2–5]. Despite enhancements in available treatment options including advances in the intensive care unit (ICU), neuraminidase inhibitor (NAI) administration and antibiotic use for concomitant or secondary bacterial infections, mortality associated with critical care admissions due to severe influenza remained high (14–22%) [2, 5] and has resulted in over 500,000 global annual deaths [6, 7].

Risk factors for severe pH1N1 influenza include diabetes, immunosuppression, pulmonary, cardiovascular, renal, hepatic, neuromuscular, hematological, or metabolic disorders, pregnancy, and other underlying comorbidities [6, 8–13]. During the H1N1 pandemic from October to December 2009, China experienced an increase in severe and critical infections. Among severe pH1N1 cases reported to the Ministry of Health, infections in patients with known risk factors for severe influenza were infrequent [14]. An epidemiologic study in Shenyang, China, showed that critical and fatal pH1N1 infections were associated with glucocorticosteroid administrations within 72 h of influenza onset [15]. Other studies of hospitalized pH1N1 patients in China, Taiwan, USA, and Korea also showed poor outcomes following glucocorticosteroid treatment [3, 16–25]. These studies gathered data from hospitalized patients and thus could not assess the broader public health impact of glucocorticosteroid use in early or uncomplicated pH1N1 influenza. Additionally, the study endpoints focused on critical or fatal cases although many additional patients required hospitalization for severe acute respiratory infection (sARI) resulting from pH1N1 infection.

Glucocorticosteroids are commonly used in China for different stages of respiratory infection management, beginning with fever control, progressing to use for pneumonia treatment, and then to management of critical pulmonary disease [26–34]. In November 2009, shortly after the Shenyang study [15], we responded to a rapid increase of sARI cases in the city of Changsha located in the Hunan Province of China. We conducted an investigation to identify sARI risk in cases of patients infected with pH1N1. In Changsha, as part of early pandemic responses, all patients with influenza-like illness (ILI) who presented to health care facilities were tested for pH1N1 infection [35] and cases of confirmed pH1N1 infection were followed closely as a precaution [35].

## Methods

### Study design and recruitment

**Fig. 1:**
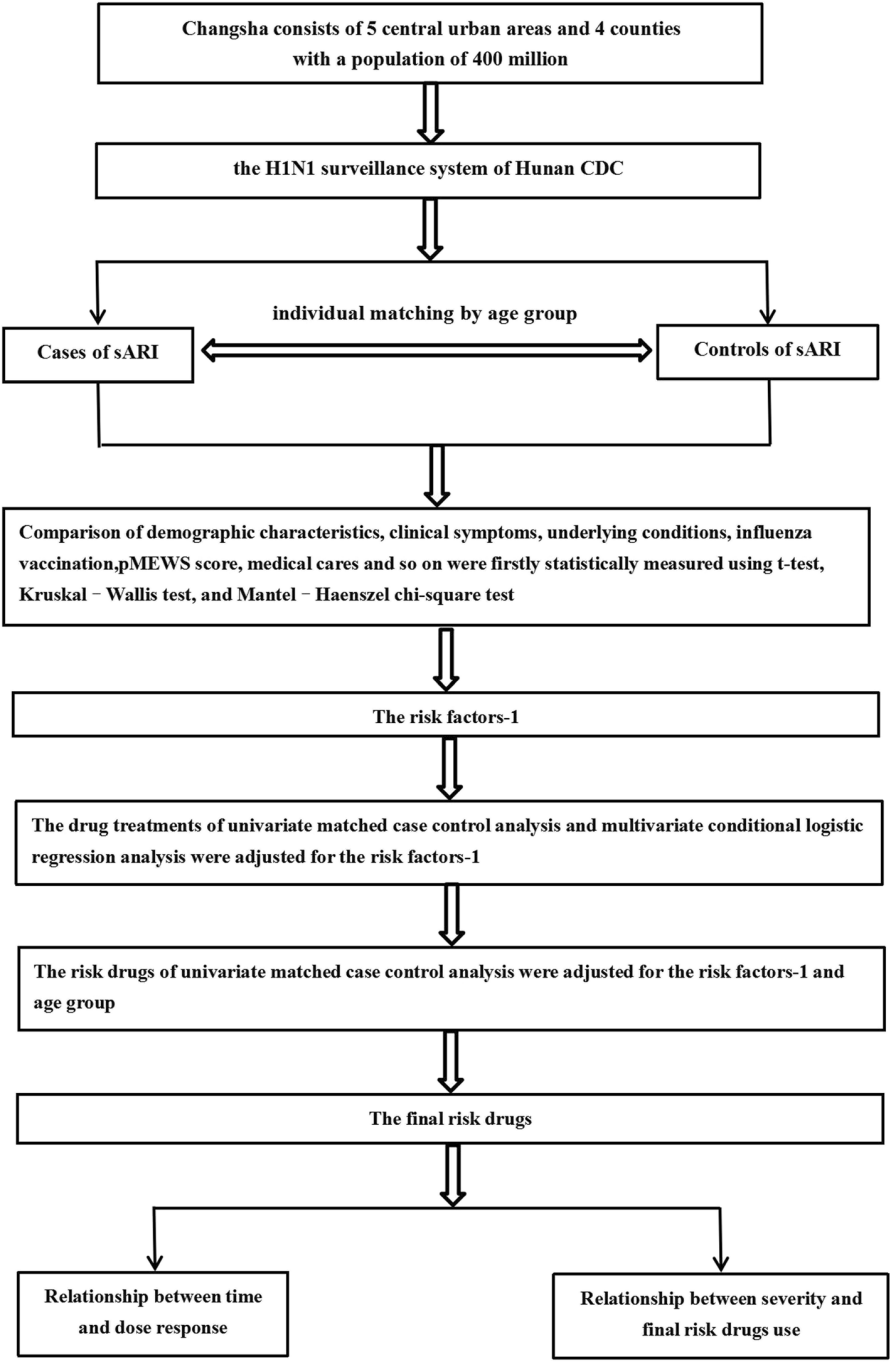
The flow chart of individual matched case-control study

We designed an individually matched case-control study enrolling patients with confirmed pH1N1 infections across the pandemic influenza surveillance in Changsha, Hunan Province, China from November to December 2009. Changsha consists of 5 central urban areas with a population of 300 million encompassing four counties surrounded by suburban and rural areas with a population of 400 million. pH1N1 infections were confirmed by laboratory RT-PCR analysis performed at the Hunan Provincial Center for Disease Control and Prevention based on published guidelines for surveillance of pH1N1 used throughout China (Fig. 1).

### Case and control definitions

Cases of sARI were defined as having ILI (fever greater than or equal 38 °C with cough or sore throat), with laboratory confirmed pH1N1 infection, and respiratory distress. Respiratory distress was defined as having oxygen saturation less than 94% regardless of age, or a resting respiratory rate > 26 times/min for cases whose age was greater than 5 years, greater than 40 times/min for children aged between 1 and 5 years, or greater than 50 times/min for children aged between 2 and 11 months. Pneumonia was defined as any infiltrate seen on a chest radiograph irrespective of sARI or respiratory distress. Critical cases were defined as sARI with any one of the following: death, respiratory failure, septic shock, multiple organ dysfunction, requiring mechanical ventilation, or ICU admission.

Controls were randomly selected from all laboratory confirmed pH1N1 cases reported through the community-based surveillance system that did not meet the sARI case definition. Controls were individually matched by ILI onset within 3 weeks of the matched sARI case and with similar age group (≤3 years, 4–12 years, 13–49 years, and ≥ 50 years) (Fig. 1).

### Study measures and comparisons

Physiological variables from the Pandemic Medical Early Warning Score (pMEWS) were used to assess patient condition [36]. These variables included systolic blood pressure, pulse rate, respiratory rate, temperature, consciousness, and blood oxygen saturation. The modified pMEWS score was determined daily using data extracted from medical files. Additionally, data on daily patient complaints and medications administered were also obtained.

sARI onset was defined as the date and time when the patient first met the sARI case criteria. Onset of pneumonia was recognized as the date and time when chest radiographs first showed infiltrates consistent with pneumonia. Critical onset was defined as the date and time that a patients first met the critical case definition criteria.

sARI cases and controls were interviewed regarding the onset of ILI, respiratory distress, underlying conditions, visits to outpatient or inpatient health care facilities, self-medication, influenza vaccination history, and contact before symptom onset with other persons that had ILI. Parents were interviewed in instances where children were less than 14 years old. Medical records from all health care facilities that the patients visited were reviewed in order to determine drug administration or prescription.

sARI cases were compared to controls by demographic characteristics, clinical symptoms, underlying conditions, influenza vaccination history, exposure to persons with ILI, and medical care or drugs used to treat ILI. We compared use of antipyretics, glucocorticosteroids, antivirals, antibiotics, and other medications before sARI onset in sARI cases and during the equivalent period of time after ILI onset in matched controls. The equivalent period for controls was determined using the days from ILI onset to sARI onset for each sARI case, and then these periods were randomly assigned to controls to determine medication use. Based on this we estimated glucocorticosteroid risk at different time periods over the course of sARI cases.

Glucocorticosteroids were used commonly as an anti-fever drug for treating influenza-like illness in many families and small hospitals of China including dexamethasone, hydrocortisone, methylprednisolone, etc. Commonly used pharmaceutical dosage forms included tablets, capsules, oral liquids, injections, etc. and the dose of the drug would vary depending on the severity of the condition (Fig. 1).

### Statistical analysis

Comparison of baseline characteristics, patient symptoms, pMEWS score before or at the time of glucocorticosteroid administration, frequency of contact with healthcare providers, and type of clinic before sARI onset for cases or assigned sARI onset for controls were statistically measured using t-test, Kruskal–Wallis test, and Mantel–Haenszel chi-square test. Conditional logistic regression was used to determine the matched odds ratios and 95% confidence intervals for all comparisons of risk factors with a *p*-value of less than 0.05 between sARI cases and controls in the univariable analysis. These measures were repeated with adjustment for age groups and underlying disease or conditions. A *p*-value of less than 0.05 was considered statistically significant. SPSS version 17.0 (IBM, Armonk, NY, USA) software was used for all the statistical analyses.

## Results

### Patients

A total of 50 sARI cases that occurred from November 20 to December 31 across any of the seven hospitals assigned to receive pH1N1 patients in Changsha were selected for this study. The median interval from onset of fever to sARI in cases was 4 days with a range of 1 to 9 days. Critical illness developed in 19 sARI cases with four deaths and the median interval from sARI onset to critical status was 2 days with a range of 0 to 7 days. Controls were selected and contacted from 100 cases in the H1N1 surveillance system. Among those selected for enrollment as controls, 21 did not respond, 12 refused to participate, 3 could not complete the interview, and 6 could not be contacted.

Underlying disease was present in 48% of sARI cases compared to 24% of controls (Table 1, *p*<0.01). Otherwise, sARI cases and controls did not significantly differ with regards to other underlying conditions, demographic characteristics, exposure to persons with ILI, or seasonal influenza vaccination rate. Before sARI onset in sARI cases and during the equivalent period after ILI onset in controls there were no differences in symptoms experienced between groups. During this period, 14% (7/50) of sARI cases and 6.3% (5/79) of controls had pulmonary infiltrates reported on chest radiograph (Fisher exact test *p* = 0.16, Table 1).

**Table 1:** Demographic characteristics, clinical symptoms, underlying conditions, influenza vaccination history, and exposure to persons with influenza-like illness (ILI) in 50 severe acute respiratory infection (sARI) cases and 79 ILI controls infected with pH1N1 influenza

Items	Variables	Number exposed		Percent exposed		*p*-value^c^
		Case (*N* = 50)	Control (*N* = 79)	Case	Control	
Demographic characteristics	Sex					0.19
	Male	30	38	60.0	48.1	
	Female	20	41	40.0	51.9	
	Age group					0.11
	<3y	7	9	14	11.4	
	4–12y	26	32	52	40.5	
	13–49y	9	30	18	38.0	
	>50y	8	8	16	10.1	
Symptoms before sARI ^a^	Fever					0.37
	37.5 °C–37.9 °C	10	27	20	34.2	
	38.0 °C–38.9 °C	25	34	50	43.0	
	39.0 °C − 39.9 °C	13	16	26	20.2	
	40.0 °C–40.9 °C	2	2	4	2.5	
	Cough	47	68	94	86.1	0.16
	Bloody sputum	1	0	2	0.0	0.39*
	Pulmonary Infiltrate	7	5	14	6.3	0.16*
Underlying conditions ^b^	Pregnant	1	2	2.0	2.5	0.67*
	Obesity	3	1	6.0	1.3	0.16*
	Smoker	7	6	14.0	7.6	0.24
	Drinks alcohol	1	1	2.0	1.3	0.63*
	Underlying disease	24	19	48.0	24	0.00
	Pulmonary	16	7	32.0	8.9	0.00
	Cardiovascular	4	2	8.0	2.5	0.16*
	Metabolic	5	1	10.0	1.3	0.03*
	Renal	4	2	8.0	2.5	0.16*
	Hepatic	4	6	8.0	7.6	0.59*
	Neoplastic	4	1	8.0	1.3	0.07*
	Neurologic	2	2	4.0	2.5	0.50*
	Immunosuppression	2	0	4.0	0.0	0.15*
	Number of underlying conditions					0.012
	1	18	15	36.0	19.0	
	2	3	4	6.0	5.1	
	≥3	3	0	6.0	0.0	
Vaccination history	Seasonal influenza vaccination in past 5 years	16	17	32.0	21.5	0.18
	Pandemic H1N1 vaccination	2	2	4.0	2.5	0.50*
Exposure to ILI before ILI onset	At home or work	26	42	52.0	53.2	0.90
	At home	14	13	28.0	16.5	0.12
	At work place	16	27	32.0	34.2	0.80

Symptoms before sARI ^a^: We applied the time interval from ILI onset to sARI onset of the case to the matched controls to impute sARI onset for control patients. Underlying conditions ^b^: According to “The Ministry of Health H1N1 flu diagnosis and treatment plan,” Underlying conditions included Pregnant, Obesity, Smoker, Drinks alcohol and Underlying disease etc., Underlying disease included Pulmonary, Cardiovascular, Metabolic, Renal, Hepatic, Neoplastic, Neurological, and Immunosuppression etc. Immunosuppression meant that the patient was taking drugs to suppress immunity or that the patient had a disease that caused immunosuppression directly. *p*-value^c^: Chi-Square test; *Fisher exact test

The mean interval from ILI onset to first clinic visit was 1.5 days for sARI cases and 1.1 days for controls (*p* > 0.10, t-test). No differences were found between sARI cases and controls with regards to the timing or type of first clinic visit for treatment of ILI (Table 2).

**Table 2:** Medical cares for influenza-like illness (ILI) of 48 severe acute respiratory infection (sARI) cases and 79 ILI controls infected with pH1N1 influenza

Variables	Number exposed		Percent exposed		Adjusted *p*-value^b^
	Case (*N*^a^ = 48)	Control (*N* = 79)	Case	Control	
Level of clinic of first visit:					0.67
Village	3	7	6.3	8.9	
Primary care	15	18	31.3	22.8	
Secondary care outpatient	3	4	6.3	5.1	
Tertiary care outpatient	24	40	50.0	50.6	
Above Tertiary care outpatient	3	10	6.3	12.7	
Saw a doctor before sARI*	48	71	100.0	89.9	0.06
Clinic visits before sARI*					0.50
1	19	39	39.6	49.4	
2	20	29	41.7	36.7	
≥3	10	11	20.8	13.9	

*N*^a^ = 48: Exclude 2 hospital acquired patients. Adjusted *p*-value^b^: adjusted for age group and underlying disease using conditional logistic regression. * We applied the time interval from ILI onset to sARI onset of the case to the matched controls to impute sARI onset for control patients

### Drug treatments

Matched case control analysis was adjusted for underlying disease. As shown in the univariate analysis, 60.0% (30/50) of sARI cases received glucocorticosteroids compared to 6.3% (5/79) of controls (OR_M-H_ = 16.7, 95% CI = 5.8–47.7), 30.0% (15/50) of sARI cases received pyrazolones compared to 7.6% (6/79) of controls (OR_M-H_ = 4.7, 95% CI = 1.6–13.7), and 74.0% (37/50) of sARI cases received NAIs compared to 8.9% (7/79) of controls (OR_M-H_ = 24.5, 95% CI = 8.7–68.4). Otherwise, sARI cases and controls did not significantly differ by use of amantidine, aibavirin, other antivirals, antibiotics, acetaminophen, ibuprofen, nimesulide, or traditional Chinese medicine interventions. Multivariate conditional logistic regression analysis based on the univariate analysis results found that glucocorticosteroid and NAI use was significantly differentt in sARI case versus control treatment (OR for glucocorticosteroids =8.2, 95% CI = 2.3–29.0; OR for NAIs =14.8, 95% CI = 4.9–45.2, Table 3).

**Table 3:** The comparison of drugs treatment used between 50 severe acute respiratory infection (sARI) cases and 79 controls infected with pH1N1 influenza

Drugs	Number exposed		Percent exposed		Matched odds ratio^a^ (95%CI)	Conditional logistic regression
	Case (*N* = 50)	Control (*N* = 79)	Case	Control		
Glucocorticosteroids^b^	30	5	60.0	6.3	16.7(5.8–47.7)	8.2(2.3–29.0)
Pyrazolones^c^	15	6	30.0	7.6	4.7(1.6–13.7)	3.3(0.8–14.6)
Neuraminidase inhibitor	37	7	74.0	8.9	24.5(8.7–68.4)	14.8(4.9–45.2)
Amantidine	3	6	6.0	7.6	0.9(0.2–3.8)	
Ribavirin	27	36	54.0	45.6	1.3(0.6–2.8)	
Other antivirals	15	32	30.0	40.5	0.6(0.2–1.3)	
Antibiotics	47	67	94.0	84.8	3.4(0.8–13.4)	1.0(0.2–5.5)
Acetaminophen	15	17	30.0	21.5	1.7(0.7–3.8)	
Ibuprofen	15	16	30.0	20.3	2.1(0.9–5.0)	2.1(0.7–7.1)
Nimesulide	9	15	18.0	19.0	1.0(0.4–2.7)	
Traditional Chinese medicine	33	59	66.0	74.7	0.5(0.2–1.2)	

^a^Odds Ratio is matched by underlying disease, ^b^dexamethasone or methyl prednisolone, ^c^aminopyrine or dipyrone

### Glucocorticosteroid and NAI treatment before sARI

Screening for significantly different variables including glucocorticosteroid and NAI use, risk was further analyzed based on whether drugs were received before sARI onset by matched cases and controls adjusted for age group and underlying disease status. This analysis showed that 34.0% (17/50) sARI cases received glucocorticosteroids before sARI onset compared to 3.8% (3/79) of controls across an equivalent time period following ILI onset (OR_M-H_ = 17.0, 95% CI = 2.1–135.0), and the OR subsequently increased from 8.2 to17.0. Two cases and one control showed infiltrates on chest radiograph when glucocorticosteroids were first administered and exclusion of these from the matched analysis resulted in an OR_M-H_ of 15.0 (95% CI 1.9–120.9, Table 4). The ratio of NAI treatment before sARI onset was very low with only 8.0% (4/50) sARI cases having received NAIs compared to 7.6% (6/79) of controls during an equivalent period after ILI onset (OR_M-H_ = 0.6, 95% CI = 0.1–3.4, Table 4). Across this same period, sARI cases did not significantly differ from controls regarding frequency of other antipyretics and medication use (Table 4). When glucocorticosteroids were first administered the median pMEWS score was 2 in both sARI cases and controls. Similarly, no individual component of the pMEWS score significantly differed between sARI cases and controls.

**Table 4:** Drugs used to treat influenza-like illness (ILI) from pH1N1 influenza, before sARI onset in 50 severe acute respiratory infection (sARI) cases and during equivalent period after ILI onset in 79 controls

Drugs	Number exposed		Percent exposed		Matched odds ratio^a^ (95%CI)
	Case (*N* = 50)	Control (*N* = 79)	Case	Control	
Glucocorticosteroids^b^ before sARI onset	17	3	34.0	3.8	17.0(2.1–135.0)
Glucocorticosteroids^b^ before both pneumonia and SARI	15	2	30.0	2.5	15.0(1.9–120.9)
Pyrazolones^c^	13	5	26.0	6.3	3.0(0.8–11.7)
Neuraminidase inhibitor	4	6	8.0	7.6	0.6(0.1–3.4)
Amantidine	3	4	6.0	5.1	0.3(0.0–2.8)
Ribavirin	21	32	42.0	41	1.3(0.5–3.2)
Other antivirals	6	11	38.8	61.2	0.5(0.1–2.5)
Antibiotics	43	63	86.0	79.7	0.8(0.2–2.6)
Acetaminophen	14	12	28.0	15.2	–
Ibuprofen	11	15	22.0	19.0	0.8(0.2–2.8)
Nimesulide	9	15	18.0	19.0	1.4(0.4–4.4)
Traditional Chinese medicine	28	51	56.0	64.6	1.6(0.7–3.4)

^a^Odds Ratio is matched by age group and underlying disease, ^b^dexamethasone or methyl prednisolone, ^c^aminopyrine or dipyrone

Among the 17 sARI cases who received glucocorticosteroids before sARI onset, 9 continued receiving them after sARI onset and of these 77.8% (7/9) developed critical illness and 3 (33%) died. Among the 8 sARI cases whose glucocorticosteroids were not continued after sARI onset, 50.0% (4/8) developed critical illness and 1 (12.5%) died 2 days after sARI onset. Among the other 33 sARI cases, 13 received glucocorticosteroids at or after sARI onset and only 30.8% (4/13) developed critical illness. Finally, of the remaining 20 sARI cases that never received glucocorticosteroids, 20.0% (4/20) developed critical illness (Table 5).

**Table 5:** The association between glucocorticosteroids treatment and critical cases in 50 severe acute respiratory infection (sARI) cases infected with pH1N1 influenza

Glucocorticosteroids^a^	Before sARI	After sARI		Critical		Critical%
yes	17	yes	9	yes	7	77.8
				no	2	22.2
		no	8	yes	4	50.0
				no	4	50.0
no	33	yes	13	yes	4	30.8
				no	6	60.0
		no	20	yes	4	20.0
				no	19	82.6

^a^dexamethasone or methyl prednisolone

We compared glucocorticosteroid treatments across different time periods over the course of disease and compared 19 critical cases with 31 non- critical cases. Before sARI onset, 57.9% (11/19) critical cases received glucocorticosteroids compared to 19.4% (6/31) of non-critical cases (OR_M-H_ = 5.7, 95% CI = 1.6–20.2, after adjusting for underlying disease). Of the 11 critical cases who received glucocorticosteroid before sARI onset, 63.6% (7/11) continued receiving them after sARI onset and the OR_M-H_ increased from 5.7 to 8.5 (Table 6). Receiving glucocorticosteroids only after sARI onset was not significantly associated with risk of developing critical illness and 21.1% (4/19) critical cases received glucocorticosteroids compared to 19.4% (6/31) of non-critical cases (OR_M-H_ = 1.1, 95% CI = 0.3–4.6, after adjusting for presence of underlying disease, Table 6).

**Table 6:** The comparison of glucocorticosteroids treatment at different time periods, between 19 critical cases and 31non- critical cases

Glucocorticosteroids^b^	Number exposed		Percent exposed		Matched odds ratio^a^ (95%CI)
	Critical case (*N* = 19)	Non-critical cases (*N* = 31)	Critical case	Non-critical case	
before sARI onset	11	6	57.9	19.4	5.7(1.6–20.2)
before sARI onset and during sARI and critical	7	2	36.8	6.5	8.3(1.6–44.4)
Only during sARI and critical	4	6	21.1	19.4	1.1(0.3–4.6)

^a^Odds Ratio is matched by underlying disease, ^b^dexamethasone or methyl prednisolone

### Glucocorticosteroid and pMEWS score

**Fig. 2:**
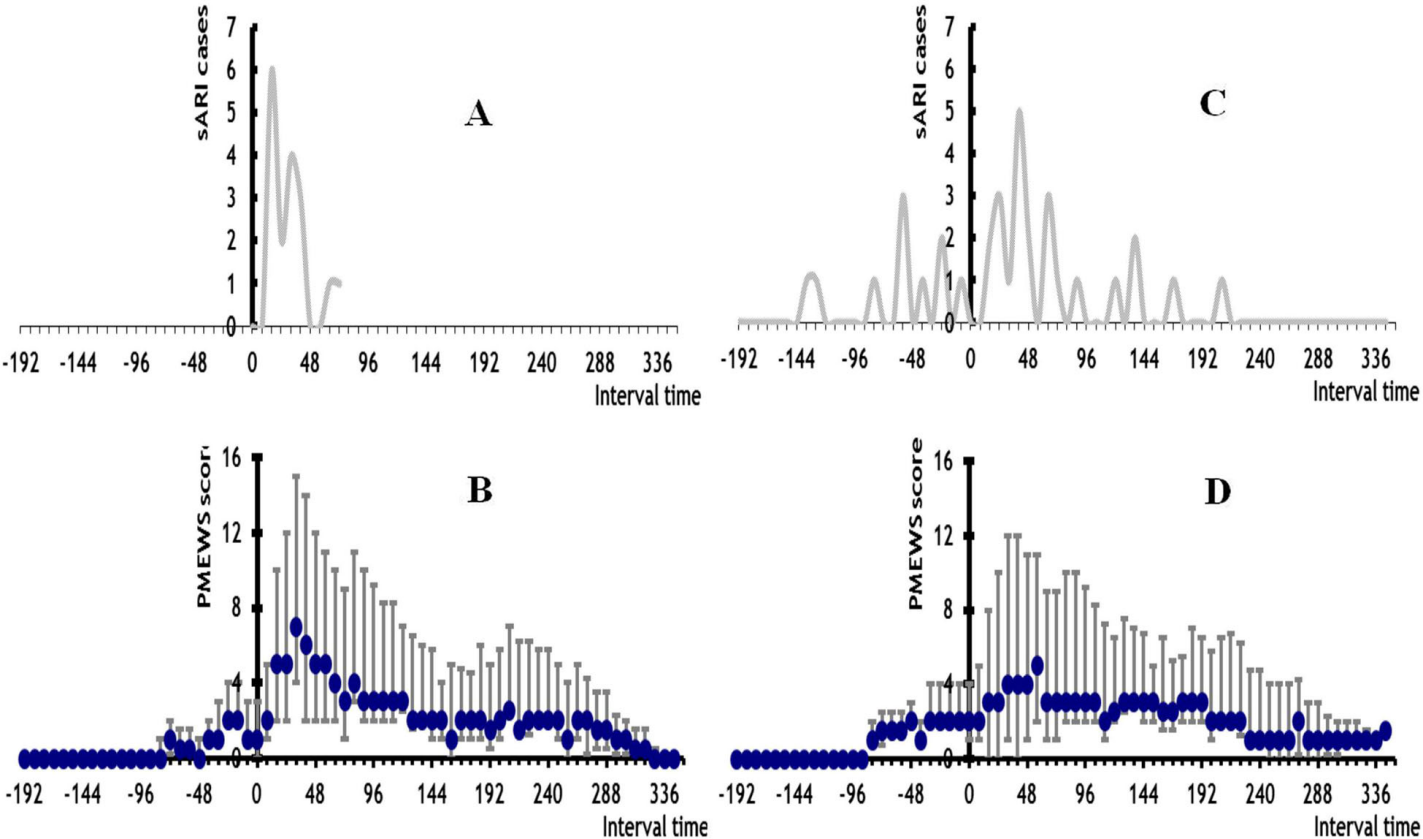
panel **a** the sARI case number of Interval from glucocorticosteroids used to sARI onset among 17 sARI patients who used the glucocorticosteroids before sARI (by 8 h), panel **b** the median pMEWS score and 75 and 25% precentile distribution among 17 sARI patients who used the glucocorticosteroids before sARI (by 8 h), panel **c** the sARI case number of Interval from equivalent assigned glucocorticosteroids use to sARI onset among 33 sARI patients who not used the glucocorticosteroids before sARI (by 8 h), panel **d** the median pMEWS score and 75 and 25% precentile distribution among 33 sARI patients who not used the glucocorticosteroids before sARI (by 8 h)

**Fig. 3:**
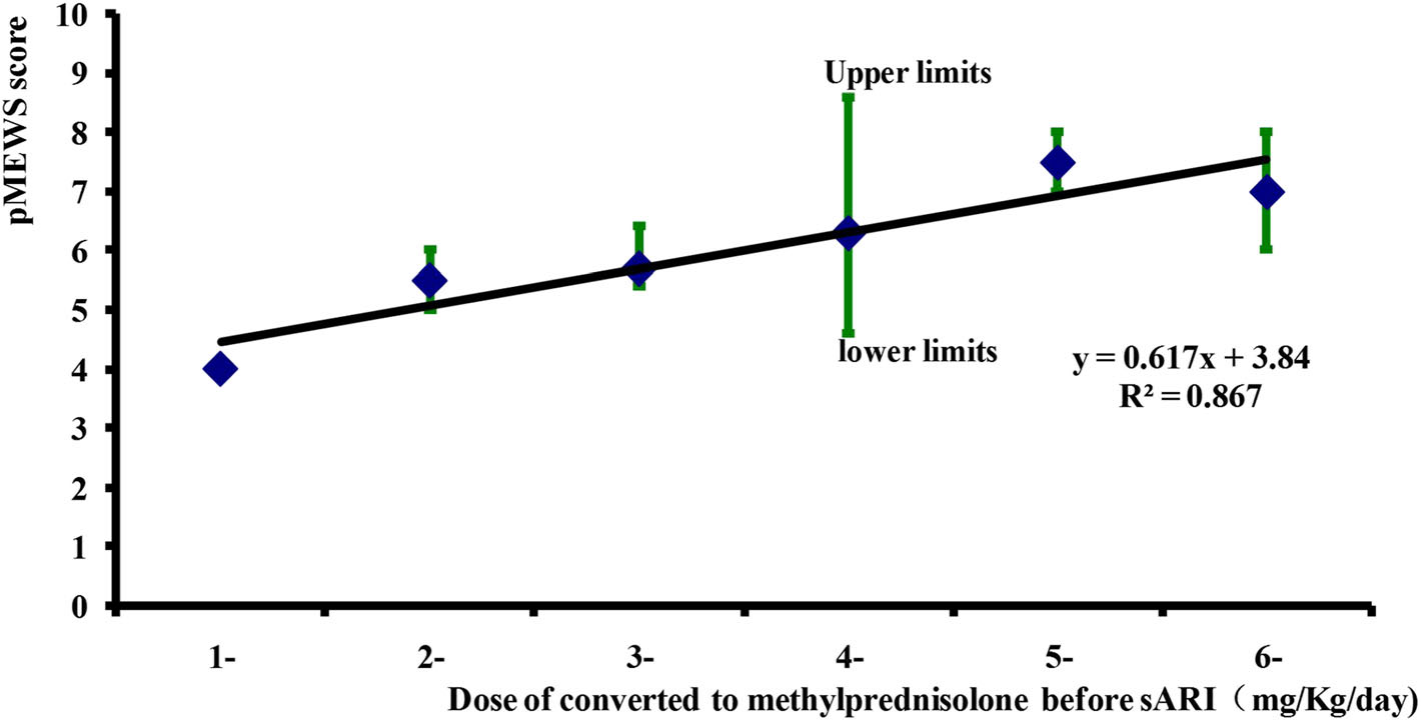
Dose response between glucocorticosteroids and median pMEWS score among 17 sARI patients who used the glucocorticosteroids before sARI

In the 17 sARI cases with glucocorticosteroid treatment before sARI onset, sARI appeared between 16 to 72 h with a peak at 24 h after administration of the first glucocorticosteroid dose (Fig. 2a). After the first glucocorticosteroid dose, the median pMEWS score quickly climbed from 1 to a maximum of 7 (interquartile range of 4–15) at 32 h (Fig. 2b). For every 1 mg/kg/day increase in glucocorticosteroid dose (methylprednisolone equivalent) prior to sARI onset, the pMEWS score at the time of sARI onset increased by 0.62 (*R*^2^ = 0.87, Fig. 3).

Imputed glucocorticosteroid initiation for the other 33 sARI cases was widely dispersed throughout the days preceding SARI onset (Fig. 2c). Among these 33 cases we observed a gradual increase in sARI onset when plotted across calendar time (Fig. 2d). After the first glucocorticosteroid dose the median pMEWS score remained at a baseline of 2 for 8 h and then climbed to a maximum of 5 (interquartile range of 2–8) at 64 h (Fig. 2d). The 5 control patients who received glucocorticosteroids showed the same pattern of rising from a median baseline score of 2 (range of 2–3) at the time of first glucocorticosteroid dose to 6 (range of 3–7) 24 h afterward.

## Discussion

The role of glucocorticosteroids for the treatment of influenza is highly controversial with some published studies showing that glucocorticosteroid use might reduce mortality and ameliorate acute lung injury induced by pH1N1virus [23, 37–46], whereas an increasing number of published studies show the opposite effect [3, 16–25]. It is reported that glucocorticosteroids may impair viral clearance and increase risk of secondary infections [47–49]. However, these studies mostly estimated the increased risk of death among patients receiving glucocorticosteroids, and the available evidence is of low quality, with a major potential concern regarding confounding by indication, and these results should be interpreted with caution [50, 51]. We conducted a case control study to identify the risk of sARI with confirmed pH1N1 infections and systematically compared the risk from receiving major potential concern before sARI onset to after sARI onset. We further assessed the correlation between disease severity and glucocorticosteroid administration using time-dependent pMEWS scores and dose responses. Our study found that receiving glucocorticosteroids before sARI onset was not only an important risk factor for developing sARI but also increased the risk of developing subsequent critical illness or death, whereas receiving glucocorticosteroid treatment only after sARI onset did not increase the risk of severe illness. A prospective, randomized clinical trial would be the best study design methodology to conclusively show whether glucocorticosteroids contribute to severe disease; however, since the incidence of severe disease is rare, a prospective study design would not be feasible. Additionally, randomized controlled clinical trials only work well in instances with high adverse outcome rates, and since there is ample documented evidence showing harmful effects of steroid administration to immune response status, these studies would be unethical. In our investigation, the percentage of controls with glucocorticosteroid exposure was only about 3%. A larger cohort of pH1N1 patients is needed to enroll adequate number of patients who used glucocorticosteroids. While all pH1N1 patients showed ILI at disease onset, the total positive pH1N1 percentage among ILI patients is high even during pandemics, further complicating the implementation of prospective randomized clinical trials.

To address potential confounding by indication, we used sARI as an endpoint as opposed to critical illness and further randomly selected controls across community mild pH1N1cases. Controls were subsequently individually matched by age group and ILI onset within 3 weeks of the sARI case. We compared glucocorticosteroid use before sARI onset in sARI cases and during the equivalent period after ILI onset in matched controls and found that early use of glucocorticosteroid to reduce fever or to prevent development of severe disease in ILI patients resulted in a 17-fold increased risk of developing sARI. This association was supported by several other findings. First, sARI developed rapidly within a narrow time window that was consistent with the biologic effect and rapid immunosuppressive action of glucocorticosteroids (Fig. 2). The association was also highly specific with no other associations found when examining use of other antipyretics or medications for ILI. Our findings were also independent of underlying diseases or conditions. The association was also supported by a dose response effect with a higher pMEWS score following higher doses of glucocorticosteroids. Second, we further compared glucocorticosteroid treatment across different time periods during the disease course of 19 critical cases and 31 non- critical cases, and found that receiving glucocorticosteroid before sARI onset increased the risk of developing subsequent critical illness or death (OR_M-H_ = 5.7, 95% CI = 1.6–20.2, after adjusting for underlying disease). The OR_M-H_ increased from 5.7 to 8.5 for continued glucocorticosteroid administration after sARI onset and we found that when glucocorticosteroid treatment was initiated after sARI onset the risk of severe illness (OR_M-H_ = 1.1, 95% CI = 0.3–4.6, after adjusting for underlying disease, Table 6) did not increase.

Other studies that assessed early use of glucocorticosteroid for fever and symptom relief estimated a much lower risk than our findings [3, 16–25]. This discrepancy can be explained in part by examining limitations in these studies. First, only hospitalized patients were used resulting in a selection bias which decreased the representation of mild ILI patients. Second, patients who received later glucocorticosteroids were included in the group that was unexposed to early glucocorticosteroids, and glucocorticosteroids given at later stages following development of severe influenza also had unfavorable outcomes. Finally, the outcome measure of these studies was critical illness or death instead of sARI. Thus, the outcome variable was more severe in these studies. The design of our investigation resulted in an estimate of risk that is referent to the community at large for sARI and is more inclusive of severe influenza-related disease. Nevertheless, many individuals with pH1N1 and mild ILI did not seek medical treatment. Thus, these cases could not receive glucocorticosteroids. Considering this possibility, the actual increase in risk of sARI from receiving glucocorticosteroid for ILI may be far higher than the 17-fold increase found in our study.

In retrospective studies of diseases that follow medical treatment, it is necessary to ensure that exposure to treatment was not precipitated by an early manifestation of severe outcomes. At the time that glucocorticosteroids were first administered, objective evidence from the pMEWS score with individual components revealed no differences between groups. Physicians themselves reported that controlling fever was the indication, but there was no difference in the temperature of sARI cases and controls when glucocorticosteroid were administered. Physicians also offered no other observed indicator of severity upon which they could based their decision to use glucocorticosteroid. Other surveys conducted by Chinese Field Epidemiology Training Program showed that glucocorticosteroids were used commonly in rural areas of China as an anti-fever drug. But the current two guidelines for glucocorticosteroids use, it is clearly stated that glucocorticosteroids should not be used for such indications. Possible reasons that these drugs were so commonly used even regardless of guidelines was due to the very quick fever reducing effect and low cost. Generally speaking, well trained specialists will only use glucocorticosteroids management of critical pneumonia patients whereas village or township practitioners administer them indiscriminately to reduce fever and provide symptomatic relief. Many patients who developed sARI while on glucocorticosteroids were fortunate that their hospital physician did not continue administration for simple ILI cases. However, there were a few unfortunate cases where continued glucocorticosteroid use may have contributed to mortality.

NAIs including oseltamivir and peramivir are used for influenza treatment, can reduce influenza symptoms, and improve survival, with increasing benefit when administered within 48 h of symptom onset [25, 52–57]. Our investigation found that 74.0% (37/50) of sARI cases received NAIs compared to only 8.9% (7/79) of controls (OR_M-H_ = 24.5, 95% CI = 8.7–68.4), however, of these 66% (33/50) sARI cases received NAIs after sARI onset. The ratio of NAI treatment before sARI onset was very low with only 8.0% (4/50) of sARI cases having received NAIs compared to 7.6% (6/79) of controls during an equivalent period after ILI onset (OR_M-H_ = 0.6, 95% CI = 0.1–3.4). These results suggest that late NAIs use probably greatly limited its effectiveness.

One limitation of this study is its retrospective design. Because the information regarding treatment and clinical indicators of severity were all derived from medical records, we were unable to control the quality of obtained information. While we conducted detailed analyses and assessed correlations between disease severity and glucocorticosteroid administration using time-dependent pMEWS scores and dose responses, we did not analyze further differences in treatments received by patients in the case versus control groups in order to determine whether administered treatments were statistically similar between the two groups.

## Conclusions

A major concern with influenza epidemics and pandemics is that hospitals will not be able to cope with the rapid increase in demand for inpatient medical services. This study has demonstrated a preventable risk factor for hospitalization during influenza epidemics. Reduction of glucocorticosteroid use would result in allowing medical facilities to cope with the sudden increases in resource utilization. We recommend establishment and implementation of strict guidelines in order to avoid inappropriate glucocorticosteroid use as well as designing interventions that would encourage doctors to change their medical prescribing habits. Similarly, government sponsored programs should be implemented to set up sARI surveillance to monitor glucocorticosteroid use among the sARI patients and efforts should be made to decrease the price of other safe anti-fever drugs.

## Data Availability

The datasets used and analyzed during the current study are available from the corresponding author on reasonable request.

## Abbreviations

ICU: Intensive care unit
ILI: Influenza-like illness
NAI: Neuraminidase inhibitor
pH1N1: Pandemic influenza A
pMEWS: Pandemic Medical Early Warning Score
sARI: Severe acute respiratory infection
